# Prognostic value of preoperative inflammatory cell-to-HDL cholesterol ratios in patients with non-metastatic clear cell renal cell carcinoma

**DOI:** 10.3389/fonc.2026.1876997

**Published:** 2026-08-12

**Authors:** Minchao Qin, Zhongwen Feng, Chao Zhang, Wei Song, Kai Wang, Zhen Chen

**Affiliations:** 1 Department of Urology, Jincheng People’s Hospital, Jincheng, Shanxi, China; 2 Department of Urology, Sir Run Run Hospital Nanjing Medical University, Nanjing, Jiangsu, China; 3 Department of Urology, The Third Affiliated Hospital of Soochow University, Changzhou, Jiangsu, China

**Keywords:** clear cell renal cell carcinoma, high-density lipoprotein cholesterol, monocyte-to-HDL-C ratio, neutrophil-to-HDL-C ratio, prognosis

## Abstract

**Background:**

To investigate the prognostic significance of preoperative inflammatory cell-to-high-density lipoprotein cholesterol (HDL-C) ratios in patients with non-metastatic clear cell renal cell carcinoma (ccRCC) undergoing surgery.

**Methods:**

A total of 308 patients with non-metastatic ccRCC who underwent radical or partial nephrectomy between 2003 and 2012 were retrospectively enrolled. Preoperative neutrophil-to-HDL-C ratio (NHR), monocyte-to-HDL-C ratio (MHR), platelet-to-HDL-C ratio (PHR), and lymphocyte-to-HDL-C ratio (LHR) were calculated from peripheral blood tests. Time-dependent ROC analyses were performed to compare the prognostic discrimination of these markers for overall survival (OS) and cancer-specific survival (CSS). Kaplan–Meier analysis and Cox proportional hazards regression were used to evaluate survival associations. The incremental prognostic value of NHR and MHR beyond clinicopathological factors was assessed using C-index and decision curve analysis (DCA).

**Results:**

Among the four biomarkers, NHR and MHR demonstrated more consistent prognostic discrimination for OS and CSS than PHR and LHR. Patients with high NHR or high MHR had significantly worse OS and CSS (all log-rank P < 0.0001). After adjustment for age, Fuhrman grade, and T stage, both ln-NHR and ln-MHR remained independently associated with poorer OS and CSS (all P < 0.001). Bootstrap resampling demonstrated stable cutoff estimates for NHR and MHR. Incorporation of NHR or MHR into clinicopathological models improved model discrimination and net benefit; however, the incremental prognostic gain was modest.

**Conclusions:**

Preoperative NHR and MHR were independent prognostic factors for OS and CSS in patients with non-metastatic ccRCC. Although they provided additional risk stratification beyond conventional clinicopathological factors, their clinical utility requires further validation in multicenter cohorts.

## Introduction

Renal cell carcinoma (RCC) is one of the most common malignancies of the urinary system, and clear cell renal cell carcinoma (ccRCC) is its predominant histological subtype, accounting for approximately 70%–80% of all RCC cases (1). Although substantial progress has been made in imaging diagnosis, surgical management, and systemic therapy in recent years, clinical outcomes among patients with ccRCC remain highly heterogeneous. Some patients achieve long-term survival after radical or partial nephrectomy, whereas others, even with a similar clinical stage, may experience early recurrence, metastasis, or cancer-related death. Therefore, identifying simple, reproducible, and clinically practical prognostic biomarkers is important for improving risk stratification, guiding individualized follow-up, and supporting decisions regarding adjuvant therapy.

At present, prognostic assessment of ccRCC mainly relies on clinicopathological models such as TNM stage (2), Fuhrman grade (3), and the Mayo SSIGN score (4). However, these parameters do not fully reflect systemic inflammatory status and metabolic alterations associated with tumor progression. Growing evidence suggests that tumor-associated inflammation plays an important role in tumor initiation, invasion, metastasis, and treatment resistance (5, 6). Peripheral blood inflammatory components, including neutrophils, monocytes, lymphocytes, and platelets, can contribute to malignant progression through multiple mechanisms, such as modulating the tumor microenvironment, promoting angiogenesis, and suppressing antitumor immunity (7). Accordingly, hematological indices derived from circulating immune cells have attracted increasing attention in recent years and have been applied to prognostic assessmen in various malignancies (8).

Dysregulated lipid metabolism has also been increasingly recognized as an important feature of ccRCC. Beyond its role in reverse cholesterol transport, reduced HDL-C levels have been reported to be associated with unfavorable outcomes in several malignancies. Available evidence suggests that lower HDL-C levels may be associated with poor outcomes in several malignancies (9). Combining circulating immune cell counts with HDL-C to construct composite ratio indices offers a way to simultaneously reflect inflammatory and metabolic status. Such composite measures may provide an integrated reflection of inflammatory and metabolic status. In recent years, the neutrophil-to-HDL-C ratio (NHR), monocyte-to-HDL-C ratio (MHR), platelet-to-HDL-C ratio (PHR), and lymphocyte-to-HDL-C ratio (LHR) have drawn growing interest for their clinical relevance in cardiovascular disease, metabolic disorders, and certain cancers (10–12). Nevertheless, their prognostic significance in ccRCC remains unclear. Moreover, systematic comparisons of their predictive performance are still lacking.

Therefore, this study aimed to systematically compare the prognostic value of NHR, MHR, PHR, and LHR in patients with surgically treated ccRCC. Furthermore, we investigated the associations of NHR and MHR with clinicopathological characteristics and survival outcomes and evaluated whether these markers could provide additional prognostic information beyond conventional clinicopathological factors.

## Materials and methods

### Study description and population

A total of 414 patients with ccRCC who underwent surgical treatment at the Third Affiliated Hospital of Soochow University between 2003 and 2012 were initially identified. The inclusion criteria were as follows: (I) histopathological confirmation of ccRCC; (II) underwent radical nephrectomy or partial nephrectomy; (III) no history of other malignancies; (IV) no preoperative neoadjuvant therapy; and (V) complete preoperative laboratory tests and clinicopathological data were available. Patients were excluded based on the following criteria: (I) concurrent acute infection, chronic inflammatory disease, hematological disorder, or autoimmune disease; (II) use of lipid-lowering medications prior to surgery; (III) missing preoperative HDL-C or other key laboratory values; and (IV) incomplete follow-up data or loss to follow-up. After the exclusion of 106 patients, a final cohort of 308 patients was enrolled in the analysis.

### Data collection

The collected clinicopathological data included age at surgery, sex, Fuhrman grade, tumor size, pathological T stage, pathological N stage, lymphovascular invasion (LVI), and tumor necrosis. Tumor staging was determined according to the 2010 American Joint Committee on Cancer (AJCC) TNM staging system. Tumor necrosis was defined as microscopic coagulative necrosis. LVI was defined as the presence of tumor cells within endothelial-lined vascular or lymphatic lumens without an identifiable muscular wall. All preoperative laboratory parameters were obtained from routine blood tests performed within one week before surgery. These included neutrophil count (×10^9/L), monocyte count (×10^9/L), platelet count (×10^9/L), lymphocyte count (×10^9/L), hemoglobin (g/L), high-density lipoprotein cholesterol (HDL-C, mmol/L), alkaline phosphatase (U/L), and lactate dehydrogenase (U/L). Complete blood counts were measured using the Sysmex XN-9000 automated hematology analyzer (Sysmex Corporation, Kobe, Japan), and serum biochemical parameters, including HDL-C, ALP, and LDH, were measured using Beckman Coulter AU5800 chemistry analyzer (Beckman Coulter Inc., Brea, CA, USA) according to the manufacturers’ standard protocols.

Based on these laboratory parameters, the following four ratio indices were calculated:

Neutrophil-to-HDL-C ratio (NHR) = neutrophil count (×10^9/L)/HDL-C (mmol/L);
Monocyte-to-HDL-C ratio (MHR) = monocyte count (×10^9/L)/HDL-C (mmol/L);
Platelet-to-HDL-C ratio (PHR) = platelet count (×10^9/L)/HDL-C (mmol/L);
Lymphocyte-to-HDL-C ratio (LHR) = lymphocyte count (×10^9/L)/HDL-C (mmol/L).

### Follow-up assessment

Follow-up information was obtained through outpatient visits, telephone interviews, and medical record system review. Overall survival (OS) was defined as the interval from the date of surgery to death from any cause or the last follow-up. Cancer-specific survival (CSS) was defined as the interval from surgery to death attributable to ccRCC or the last follow-up. During the follow-up period, 40 patients experienced all-cause mortality for OS analysis, and 36 patients experienced cancer-specific mortality for CSS analysis.

### Time-dependent ROC analysis and cutoff value determination

Time-dependent receiver operating characteristic ROC curve analysis was performed to evaluate the predictive performance of NHR, MHR, PHR, and LHR for OS and CSS at 1-, 3-, and 5-year time points. The area under the curve was used to compare the discriminative ability of each indicator. Based on their relatively superior and stable predictive performance, NHR and MHR were selected for subsequent Kaplan–Meier survival analysis and further prognostic evaluation. The optimal cutoff values of NHR and MHR for survival stratification were determined using the maximally selected rank statistics method, yielding cutoff values of 4.52 and 0.49, respectively. Because NHR and MHR showed skewed distributions, logarithmic transformations were applied before Cox regression analyses, and the transformed variables were analyzed as ln-NHR and ln-MHR. Internal validation was performed using bootstrap resampling with 1, 000 repetitions. The stability of optimal cutoff values and hazard estimates was evaluated by calculating bootstrap-derived medians and 95% confidence intervals.

### Statistical analysis

Continuous variables are presented as medians with interquartile ranges (IQRs), and categorical variables as numbers and percentages. Differences between groups stratified by NHR or MHR were compared using the Mann–Whitney U test, chi-square test, or Fisher’s exact test, as appropriate. Time-dependent ROC analysis was performed to assess the predictive ability of NHR, MHR, PHR, and LHR for OS and CSS. Based on their relatively better and more stable performance, NHR and MHR were selected for further analyses. Optimal cutoff values for NHR and MHR were determined using maximally selected rank statistics. Kaplan–Meier curves were generated according to these cutoff values, and survival differences were compared using the log-rank test. Because NHR and MHR were not normally distributed, both variables were log-transformed before Cox regression analyses. Univariable Cox proportional hazards regression analyses were performed to evaluate the associations between candidate variables and OS or CSS. Considering the limited number of survival events and to minimize the risk of model overfitting, multivariable Cox regression models were adjusted for clinically established prognostic factors, including age, Fuhrman grade, and T stage, together with ln-NHR or ln-MHR. Results are reported as hazard ratios (HRs) with 95% confidence intervals (CIs). To evaluate the added prognostic value of NHR and MHR, Cox-based models were constructed using clinicopathological predictors alone or in combination with ln-NHR or ln-MHR. Model discrimination was assessed using the C-index, and clinical utility was evaluated by decision curve analysis. All analyses were performed using R software version 4.4.0. A two-sided *P <* 0.05 was considered statistically significant.

## Results

### Baseline clinicopathological characteristics

**Figure 1:**
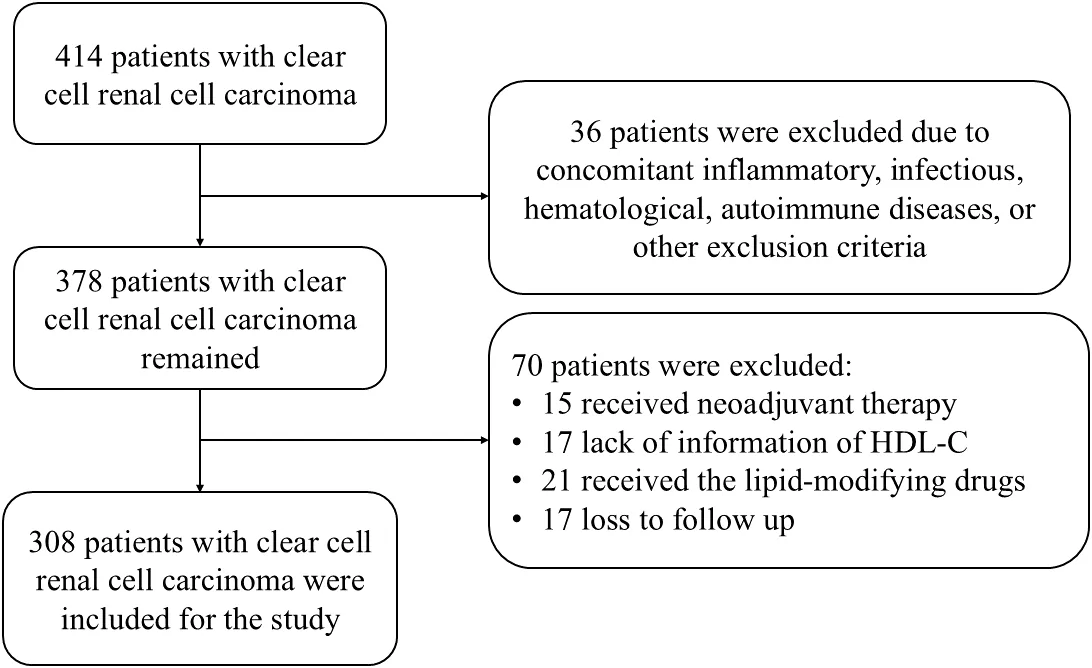
Flow diagram of the study selection process. A total of 308 patients with non-metastatic clear cell renal cell carcinoma who underwent radical or partial nephrectomy between 2003 and 2012 were included after applying the predefined inclusion and exclusion criteria.

A total of 414 patients with ccRCC who underwent radical or partial nephrectomy were initially screened. After applying the predefined inclusion and exclusion criteria, 308 patients were finally included in this study (**Figure 1**). Their baseline clinicopathological characteristics were shown in **Table 1**. There were 193 men (62.7%), and 160 patients (51.9%) were younger than 60 years. Fuhrman grade 2 was the most common grade (51.7%). Most patients had pathologic T1a (48.1%) or T1b disease (33.8%). Lymph node metastasis (N1) was observed in 10 patients (3.2%). LVI and tumor necrosis were observed in 18 (5.8%) and 27 (8.8%) patients, respectively. The median tumor size was 4.20 cm (interquartile range [IQR], 3.00–6.00). The median values of NHR, MHR, PHR, and LHR were 3.05, 0.37, 165.49, and 1.36, respectively. The median levels of HDL-C, LDL-C, and TG were 1.16 mmol/L, 2.37 mmol/L, and 1.63 mmol/L, respectively.

**Table 1:** Baseline clinicopathological characteristics.

Variable	Overall (n = 308)
Sex, n (%)	
Male	193 (62.7)
Female	115 (37.3)
Age, years, n (%)	
<60	160 (51.9)
≥60	148 (48.1)
Fuhrman grade, n (%)	
1	64 (21.5)
2	154 (51.7)
3	63 (21.1)
4	17 (5.7)
Lymphovascular invasion, n (%)	
No	290 (94.2)
Yes	18 (5.8)
Tumor size, cm, median [IQR]	4.20 [3.00, 6.00]
T stage, n (%)	
T1a	148 (48.1)
T1b	104 (33.8)
T2	32 (10.4)
T3–4	24 (7.8)
N stage, n (%)	
N0	298 (96.8)
N1	10 (3.2)
Tumor necrosis, n (%)	
No	281 (91.2)
Yes	27 (8.8)
ALP, U/L, median [IQR]	75.50 [60.00, 91.00]
LDH, U/L, median [IQR]	147.00 [130.25, 166.00]
HGB, g/L, median [IQR]	136.00 [125.00, 147.00]
HDL-C, mmol/L, median [IQR]	1.16 [0.99–1.41]
LDL-C, mmol/L, median [IQR]	2.37 [1.96–2.89]
TG, mmol/L, median [IQR]	1.63 [1.22–2.39]
NHR, median [IQR]	3.05 [2.24, 4.15]
PHR, median [IQR]	165.49 [126.45, 223.09]
MHR, median [IQR]	0.37 [0.28, 0.50]
LHR, median [IQR]	1.36 [1.13, 1.83]

ALP, alkaline phosphatase; LDH, lactate dehydrogenase; HGB, hemoglobin; HDL-C, high-density lipoprotein cholesterol; LDL-C, low-density lipoprotein cholesterol; TG, triglycerides; NHR, neutrophil-to-high-density lipoprotein cholesterol ratio; MHR, monocyte-to-high-density lipoprotein cholesterol ratio; PHR, platelet-to-high-density lipoprotein cholesterol ratio; LHR, lymphocyte-to-high-density lipoprotein cholesterol ratio; IQR, interquartile range.

### Time-dependent ROC analysis

**Figure 2:**
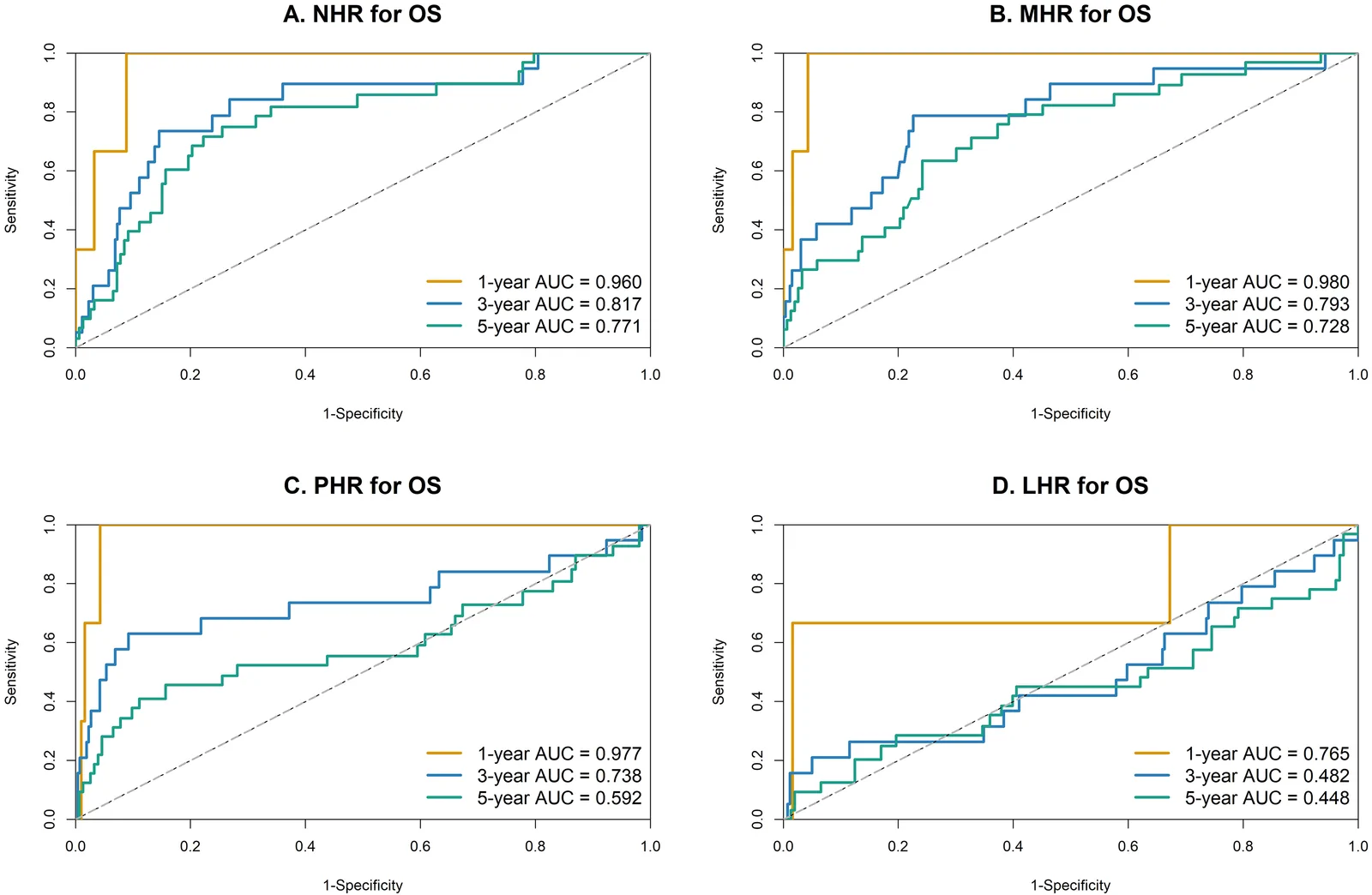
Time-dependent receiver operating characteristic (ROC) curves of the neutrophil-to-high-density lipoprotein cholesterol ratio (NHR), monocyte-to-high-density lipoprotein cholesterol ratio (MHR), platelet-to-high-density lipoprotein cholesterol ratio (PHR), and lymphocyte-to-high-density lipoprotein cholesterol ratio (LHR) for predicting overall survival (OS). **(A)** NHR; **(B)** MHR; **(C)** PHR; and **(D)** LHR. The predictive performance of each biomarker was evaluated using time-dependent ROC analysis at 1, 3, and 5 years after surgery, and the corresponding area under the curve (AUC) values are presented. Because only three OS events occurred within the first postoperative year, the 1-year AUC estimates should be interpreted with caution.

**Figure 3:**
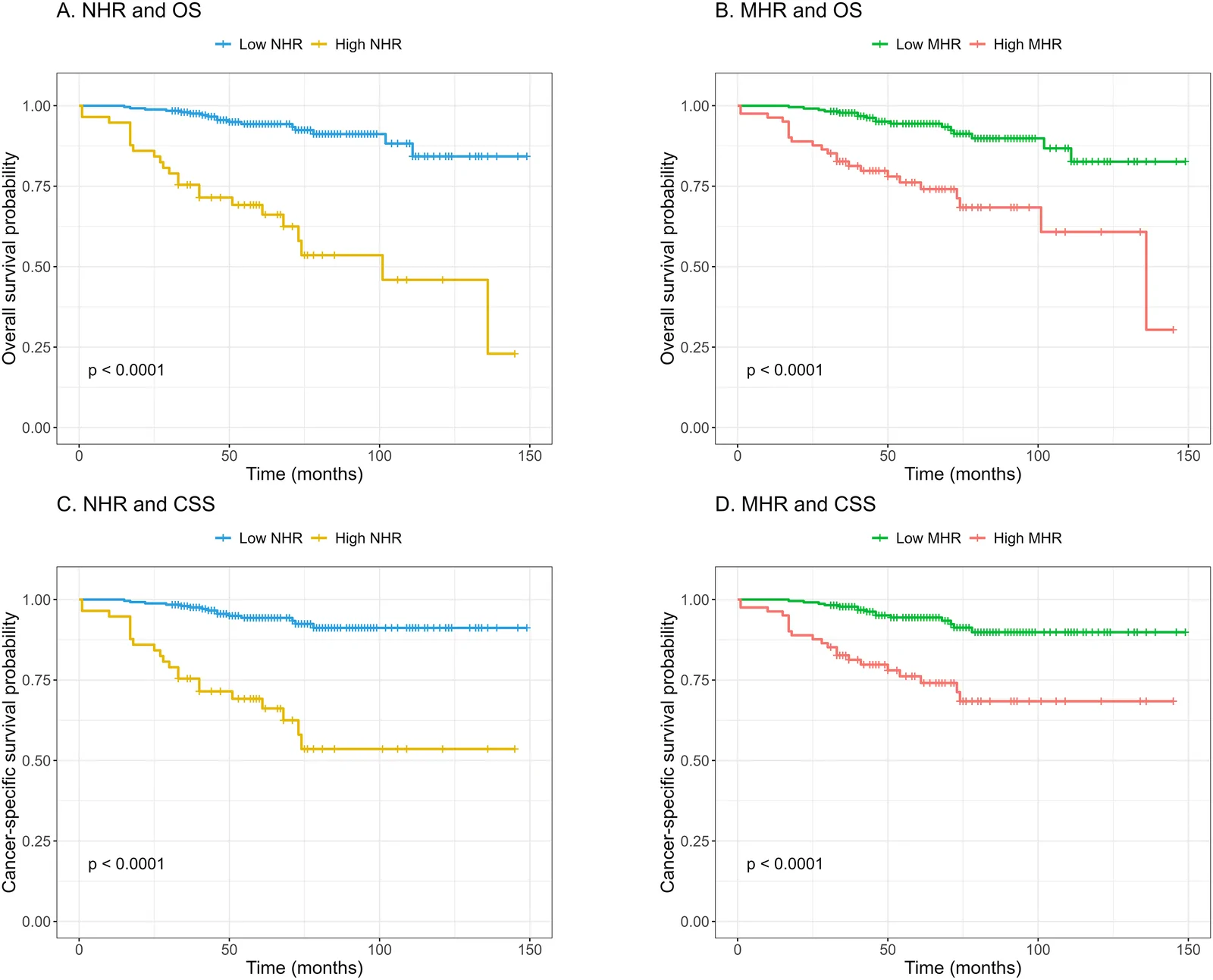
Kaplan–Meier survival curves according to NHR and MHR. **(A)** Overall survival (OS) according to NHR; **(B)** OS according to MHR; **(C)** cancer-specific survival (CSS) according to NHR; and **(D)** CSS according to MHR. Patients were classified into high- and low-level groups based on the optimal cutoff values determined by maximally selected rank statistics (NHR = 4.52; MHR = 0.49). Survival differences were assessed using Kaplan–Meier analysis and compared with the log-rank test. Patients with high NHR or high MHR levels showed significantly poorer OS and CSS than those with low levels (all log-rank *P* < 0.0001).

Time-dependent ROC analysis was performed to compare the prognostic performance of NHR, MHR, PHR, and LHR for predicting OS (**Figure 2**). During the first postoperative year, only three OS events were observed; therefore, the corresponding 1-year AUC estimates should be interpreted with caution. The AUCs of NHR at 1, 3, and 5 years were 0.960, 0.817, and 0.771, respectively, whereas those of MHR were 0.980, 0.793, and 0.728. In comparison, the corresponding AUCs for PHR were 0.977, 0.738, and 0.592, whereas those for LHR were 0.765, 0.482, and 0.448. Overall, NHR and MHR demonstrated more consistent discriminatory performance than PHR and LHR, particularly at the 3- and 5-year follow-up time points. Although NHR showed slightly higher AUC values than MHR, both markers demonstrated stable discrimination across follow-up periods and were therefore selected for further analyses. The optimal cutoff values determined using maximally selected rank statistics were 4.52 for NHR and 0.49 for MHR, respectively. Patients were subsequently classified into high- and low-NHR or MHR groups according to these cutoff values for Kaplan–Meier survival analyses (**Figure 3**).

### Kaplan–Meier survival analysis according to NHR and MHR

Kaplan–Meier survival analyses based on the optimized cutoff values demonstrated that patients with high NHR had significantly worse OS and CSS than those with low NHR (both log-rank *P* < 0.0001; **Figures 3A, C**). Likewise, patients with high MHR had significantly worse OS and CSS than those with low MHR (both log-rank *P* < 0.0001; **Figures 3B, D**).

### Associations of NHR and MHR with clinicopathological characteristics

The associations of NHR and MHR with clinicopathological characteristics are presented in **Table 2** and **Table 3**. Patients in the high-NHR group were more likely to be male (77% vs. 59%, *P =* 0.018), and had a higher frequency of LVI (18% vs. 3.2%, *P <* 0.001), larger tumor size (median, 5.00 vs. 4.00 cm, *P =* 0.007), more advanced T stage (*P <* 0.001), tumor necrosis (19% vs. 6.4%, *P =* 0.007), and higher ALP levels (median, 77.00 vs. 74.00 U/L, *P =* 0.045). HGB levels tended to be lower in the high-NHR group, although the difference did not reach statistical significance (median, 132.00 vs. 137.00 g/L, *P =* 0.050). No significant differences were observed in age, Fuhrman grade, N stage, or LDH levels. Similarly, patients in the high-MHR group had a higher proportion of male patients (82% vs. 56%, *P* < 0.001), a higher proportion of grade 3–4 tumors (37% vs. 23%, *P* = 0.026), more advanced T stage (*P* < 0.001), and a higher incidence of lymph node metastasis (7.3% vs. 1.8%, *P* = 0.025) compared with those in the low-MHR group. In contrast, age, LVI, tumor size, tumor necrosis, ALP, LDH, and HGB did not differ significantly between the two groups.

**Table 2:** Clinicopathological characteristics according to the NHR group.

Variable	Low NHR (n = 251)	High NHR (n = 57)	*p*-value
Sex			0.018
Male	149 (59%)	44 (77%)	
Female	102 (41%)	13 (23%)	
Age, years	59.00 [51.00, 65.00]	59.00 [52.00, 66.00]	0.5
Fuhrman grade			0.130
1-2	183 (75%)	10 (19%)	
3-4	61 (25%)	19 (35%)	
LVI	8 (3.2%)	10 (18%)	<0.001
Tumor size, cm	4.00 [3.00, 6.00]	5.00 [3.55, 8.00]	0.007
T stage			<0.001
T1a	128 (51%)	20 (35%)	
T1b-T2	113 (45%)	23 (40%)	
T3-4	10 (4.0%)	14 (25%)	
N stage			0.093
N0	245 (98%)	53 (93%)	
N1	6 (2.4%)	4 (7.0%)	
Tumor necrosis	16 (6.4%)	11 (19%)	0.007
ALP, U/L	74.00 [58.00, 88.00]	77.00 [65.00, 109.00]	0.045
LDH, U/L	148.00 [131.00, 166.00]	146.00 [124.00, 180.00]	0.700
HGB, g/L	137.00 [126.00, 147.00]	132.00 [110.00, 147.00]	0.050

Data was presented as n (%) or median [Q1, Q3].

P values were calculated using Pearson’s chi-squared test or Wilcoxon rank-sum test, as appropriate. NHR, neutrophil-to-high-density lipoprotein cholesterol ratio; LVI, lymphovascular invasion; ALP, alkaline phosphatase; LDH, lactate dehydrogenase; HGB, hemoglobin.

**Table 3:** Clinicopathological characteristics according to the MHR group.

Variable	Low MHR (n = 226)	High MHR (n = 82)	*p*-value
Sex			<0.001
Male	126 (56%)	67 (82%)	
Female	100 (44%)	15 (18%)	
Age, years	58.00 [50.00, 64.00]	61.50 [53.00, 67.00]	0.057
Fuhrman grade			0.026
1-2	168 (77%)	50 (63%)	
3-4	51 (23%)	29 (37%)	
LVI	10 (4.4%)	8 (9.8%)	0.100
Tumor size, cm	4.00 [3.00, 6.00]	4.50 [3.00, 7.00]	0.130
T stage			<0.001
T1a	113 (50%)	35 (43%)	
T1b-T2	100 (44%)	36 (44%)	
T3-4	13 (5.8%)	11 (13%)	
N stage			0.025
N0	222 (98%)	76 (93%)	
N1	4 (1.8%)	6 (7.3%)	
Tumor necrosis	17 (7.5%)	10 (12%)	0.300
ALP, U/L	76.00 [61.00, 90.00]	73.50 [55.00, 93.00]	0.900
LDH, U/L	148.00 [133.00, 168.00]	144.50 [124.00, 164.00]	0.200
HGB, g/L	136.00 [126.00, 147.00]	136.50 [119.00, 147.00]	0.300

Data was presented as n (%) or median [Q1, Q3].

*P* values were calculated using Pearson’s chi-squared test or Wilcoxon rank-sum test, as appropriate. MHR, monocyte-to-high-density lipoprotein cholesterol ratio; LVI, lymphovascular invasion; ALP, alkaline phosphatase; LDH, lactate dehydrogenase; HGB, hemoglobin.

### Univariate Cox regression analyses for OS and CSS

The results of the univariate Cox regression analyses are shown in **Table 4**. For both OS and CSS, older age, Fuhrman grade 3–4 (vs. 1–2), positive LVI, larger tumor size, advanced T stage, N1 stage, tumor necrosis, as well as higher ln-NHR, and ln-MHR were significantly associated with worse prognosis (all *P <* 0.05). Both ln-NHR and ln-MHR showed significant associations with OS and CSS and were therefore further evaluated in multivariable Cox regression analyses. This suggested that elevated NHR and MHR may serve as important prognostic indicators in clear cell renal cell carcinoma.

**Table 4:** Univariable Cox regression analyses for OS and CSS.

Variables	OS		CSS	
	HR (95% CI)	P value	HR (95% CI)	P value
Sex		0.109		0.117
Female vs. male	0.557 (0.272–1.140)		0.547 (0.257–1.164)	
Age	1.035 (1.003–1.068)	0.034	1.034 (1.001–1.069)	0.043
Fuhrman grade		<0.001		<0.001
3–4 vs. 1–2	3.427 (1.828–6.425)		3.667 (1.890–7.115)	
LVI		<0.001		<0.001
Yes vs. no	7.738 (3.649–16.408)		8.481 (3.959–18.166)	
Tumor size	1.226 (1.123–1.339)	<0.001	1.248 (1.140–1.366)	<0.001
T stage		<0.001		<0.001
T1a	Reference		Reference	
T1b-T2	4.871 (1.806–13.142)	0.002	6.751 (1.988–22.924)	0.002
T3-4	38.278 (13.546–108.167)	<0.001	55.964 (16.113–194.377)	<0.001
N stage		<0.001		<0.001
N1 vs. N0	8.023 (3.331–19.321)		8.023 (3.331–19.321)	<0.001
Tumor necrosis		<0.001		
Yes vs. no	3.971 (1.968–8.013)		4.736 (2.326–9.641)	
ln-NHR	6.850 (3.501–13.402)	<0.001	7.608 (3.731–15.511)	<0.001
ln-MHR	6.667 (3.438–12.928)	<0.001	7.178 (3.591–14.347)	<0.001

Variables that remained statistically significant in multivariable Cox regression analyses were pathological T stage and ln-NHR, which were independently associated with both overall survival and cancer-specific survival. OS, overall survival; CSS, cancer-specific survival; HR, hazard ratio; CI, confidence interval; LVI, lymphovascular invasion; NHR, neutrophil-to-high-density lipoprotein cholesterol ratio; MHR, monocyte-to-high-density lipoprotein cholesterol ratio.

### Multivariable Cox regression analysis for OS and CSS

Multivariable Cox regression models were constructed by adjusting for age, Fuhrman grade, and T stage, with ln-NHR or ln-MHR entered separately, as shown in **Table 5** and **Table 6**. In the ln-NHR-based model, higher ln-NHR was significantly associated with poorer OS (OS: HR = 4.567, 95% CI: 2.271–9.186, *P* < 0.001) and cancer-specific mortality (CSS: HR = 4.292, 95% CI: 2.018–9.132, *P* < 0.001) (**Table 5**). Similarly, in the ln-MHR-based model, elevated ln-MHR independently predicted poorer survival outcomes, with an increased risk of OS (HR = 4.331, 95% CI: 2.330–8.050, *P* < 0.001) and CSS (HR = 4.085, 95% CI: 2.128–7.843, *P* < 0.001) (**Table 6**). Furthermore, T stage remained a significant independent prognostic factor in both models. Compared with patients with T1a disease, those with T1b–T2 and T3–4 stages exhibited significantly increased risks of all-cause and cancer-specific mortality. These findings suggest that elevated log-transformed NHR and MHR levels are independently associated with unfavorable survival outcomes in patients with ccRCC. Bootstrap validation demonstrated good stability of the data-driven cutoff values. The median bootstrap-derived thresholds were identical to the original cutoffs for both NHR (original cutoff: 4.52; bootstrap median: 4.52; 95% CI: 4.00–5.13) and MHR (original cutoff: 0.49; bootstrap median: 0.49; 95% CI: 0.34–0.69) (**Supplementary Table 1**).

**Table 5:** Multivariable Cox regression analysis including ln-NHR for OS and CSS.

Variables	OS		CSS	
	HR (95% CI)	P value	HR (95% CI)	P value
Age	1.02 (0.988–1.052)	0.224	1.018 (0.986–1.051)	0.273
Fuhrman grade		0.245		0.303
1–2	Reference		Reference	
3–4	1.556 (0.739-3.280)		1.516 (0.686-3.347)	
T stage		<0.001		<0.001
T1a	Reference		Reference	
T1b-T2	4.399 (1.560-12.405)	0.005	5.557 (1.610-19.183)	0.007
T3-4	17.136 (5.109-57.474)	<0.001	22.698(5.616-91.740)	<0.001
ln-NHR	4.567 (2.271-9.186)	<0.001	4.292 (2.018-9.132)	<0.001

OS, overall survival; CSS, cancer-specific survival; HR, hazard ratio; CI, confidence interval; ln-NHR, natural logarithm-transformed neutrophi-to-high-density lipoprotein cholesterol ratio.

**Table 6:** Multivariable Cox regression analysis including ln-MHR for OS and CSS.

Variables	OS		CSS	
	HR (95% CI)	P value	HR (95% CI)	P value
Age	1.017 (0.983-1.053)	0.330	1.014 (0.979–1.050)	0.444
Fuhrman grade		0.414		0.489
3–4 vs. 1–2	1.360 (0.650–2.849)		1.319 (0.602–2.892)	
T stage		0.003		<0.001
T1a	Reference		Reference	
T1b-T2	4.309 (1.557-11.929)	0.005	5.750 (1.670-19.799)	0.006
T3-4	22.303(6.810-73.042)	<0.001	30.852(7.861–121.089)	<0.001
ln-MHR	4.331 (2.330-8.050)	<0.001	4.085 (2.128–7.843)	<0.001

OS, overall survival; CSS, cancer-specific survival; HR, hazard ratio; CI, confidence interval; LVI, lymphovascular invasion; ln-MHR, natural logarithm-transformed monocyte-to-high-density lipoprotein cholesterol ratio.

### Supplementary analysis of the combined NHR–MHR grouping

To further evaluate the prognostic value of the combination of NHR and MHR, patients were classified into four groups according to NHR and MHR levels. Kaplan–Meier analysis showed significant differences in both OS and CSS among the four groups (log-rank *P* < 0.0001; **Supplementary Figure 1**). Patients with high levels of both NHR and MHR generally showed the poorest survival outcomes, whereas those with low levels of both markers had the most favorable prognosis. In univariable Cox regression analyses, compared with the low NHR + low MHR group, the high NHR + low MHR group and high NHR + high MHR group were associated with significantly increased risks of OS and CSS (all *P* < 0.001). After adjustment for age, Fuhrman grade, and T stage, combined NHR–MHR grouping remained independently associated with survival outcomes. The high NHR + high MHR group exhibited the highest risk of mortality for both OS (HR = 7.20, 95% CI: 3.25–15.96, *P* < 0.001) and CSS (HR = 6.86, 95% CI: 2.88–16.31, *P* < 0.001) (**Supplementary Table 2**, **3**). These findings suggest that combined NHR–MHR stratification may provide additional risk stratification and help identify patients with particularly unfavorable survival outcomes.

### Incremental prognostic value of continuous NHR and MHR

**Figure 4:**
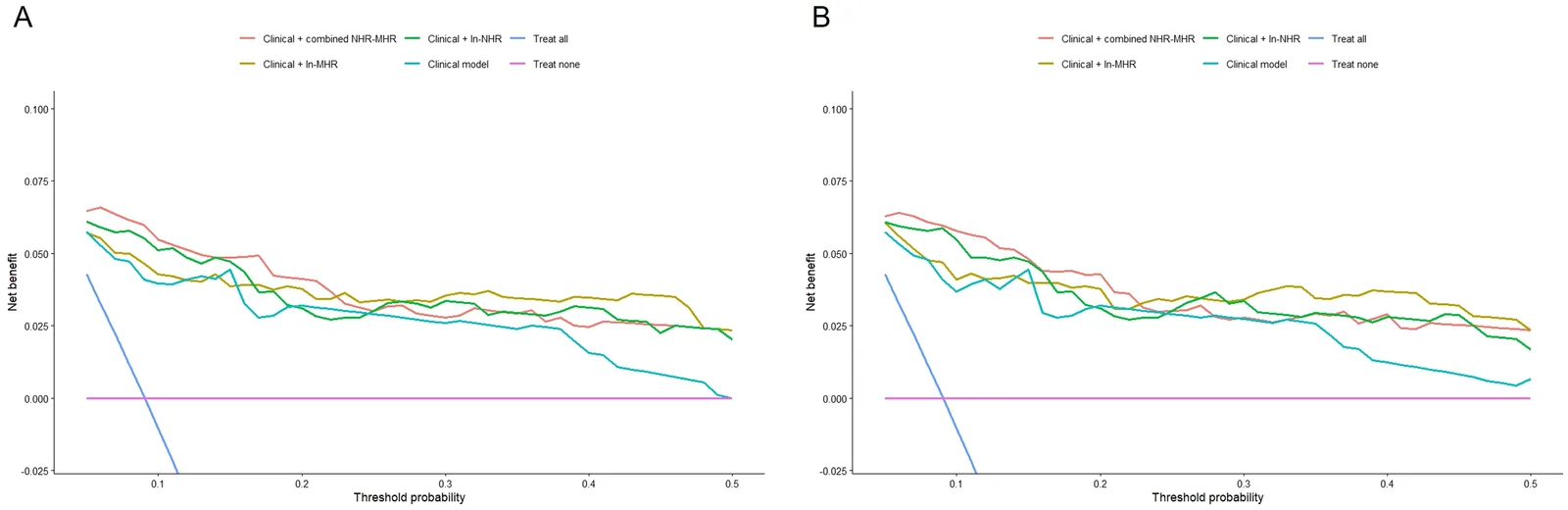
Decision curve analysis (DCA) of prognostic models for 5-year survival outcomes. **(A)** Overall survival. **(B)** Cancer-specific survival. The clinical model included age, Fuhrman grade, and T stage. Extended models were constructed by incorporating log-transformed NHR (ln-NHR), log-transformed MHR (ln-MHR), or combined NHR–MHR grouping into the clinical model. Net benefit was evaluated across threshold probabilities ranging from 5% to 50% to compare the clinical utility of different prognostic models.

To assess the incremental prognostic value of NHR and MHR beyond conventional clinicopathological factors, Cox-based models were constructed and compared (**Table 7**). The clinical model included age, Fuhrman grade, and T stage. Extended models were developed by incorporating ln-NHR, ln-MHR, or combined NHR–MHR grouping into the clinical model. For OS, the C-index increased from 0.829 in the clinical model to 0.867, 0.859, and 0.875 after adding ln-NHR, ln-MHR, and combined NHR–MHR grouping, respectively. For CSS, the corresponding C-indices increased from 0.833 to 0.868, 0.863, and 0.879, respectively. All extended models showed significantly improved model fit compared with the clinical model (likelihood ratio test, all *P* < 0.001), with the combined NHR–MHR model demonstrating the lowest AIC values. Decision curve analysis showed that models incorporating NHR or MHR provided higher net benefits than the clinical model across most threshold probabilities (**Figure 4**). The combined NHR–MHR model showed comparable or slightly higher net benefit than models incorporating individual biomarkers across several threshold probabilities. These findings suggest that incorporating NHR–MHR classification may provide additional prognostic stratification beyond conventional clinicopathological factors.

**Table 7:** Incremental prognostic value of continuous NHR/MHR and combined NHR–MHR models beyond clinical factors for OS and CSS.

Outcome	Model	n	Events	C-index	AIC	Likelihood ratio test vs. clinical model
OS	Clinical model	308	38	0.829	347.30	Reference
	Clinical model + ln-NHR	308	38	0.867	330.87	<0.001
	Clinical model + ln-MHR	308	38	0.859	329.01	<0.001
	Clinical model + combined NHR–MHR group	308	38	0.875	326.64	<0.001
CSS	Clinical model	308	34	0.833	320.12	Reference
	Clinical model + ln-NHR	308	34	0.868	307.36	<0.001
	Clinical model + ln-MHR	308	34	0.863	305.18	<0.001
	Clinical model + combined NHR–MHR group	308	34	0.879	303.36	<0.001

OS, overall survival; CSS, cancer-specific survival; NHR, neutrophil-to-high-density lipoprotein cholesterol ratio; MHR, monocyte-to-high-density lipoprotein cholesterol ratio; ln-NHR, natural logarithm-transformed neutrophil-to-high-density lipoprotein cholesterol ratio; ln-MHR, natural logarithm-transformed monocyte-to-high-density lipoprotein cholesterol ratio; C-index, concordance index; AIC, Akaike information criterion; LRT, likelihood ratio test.

## Discussion

This study systematically evaluated the prognostic value of four circulating inflammatory cell-to-high-density lipoprotein cholesterol ratios for OS and CSS in patients with ccRCC undergoing partial or radical nephrectomy. Among these biomarkers, NHR and MHR demonstrated more consistent prognostic discrimination than PHR and LHR. After adjustment for conventional clinicopathological factors, both ln-NHR and ln-MHR remained independently associated with unfavorable survival outcomes. These findings suggest that NHR and MHR may serve as potential prognostic indicators for risk stratification in patients with ccRCC.

Clear cell renal cell carcinoma exhibits marked biological heterogeneity. Tumor progression is driven by both intrinsic tumor cell behavior and host-related factors, including systemic inflammation, immune dysregulation, and metabolic reprogramming. Increasing evidence indicated that systemic inflammation contributes to ccRCC progression and patient outcomes (13). Inflammation promotes tumor progression both locally and systemically. The resulting changes in circulating inflammatory cells and mediators are readily captured by peripheral blood parameters (14, 15). Elevated neutrophil and monocyte counts have been linked to poor prognosis in various cancers, including hepatocellular carcinoma, gastric cancer, and lung cancer (16). Neutrophilia has been specifically linked to poor outcomes in cervical cancer, prostate cancer, and several other tumor types (17, 18). In patients with advanced metastatic renal cell carcinoma, elevated peripheral neutrophil counts independently predict shorter overall survival (19). Tumor cell-associated transcription factors can induce the secretion of various cytokines that drive neutrophil recruitment and raise peripheral neutrophil levels (20–22). Neutrophils promote tumor progression through multiple mechanisms. They release reactive oxygen species that induce DNA damage, and they secrete inflammatory mediators and pro-angiogenic factors that drive tumor proliferation and metastasis (23, 24). Monocytes recruited into the tumor microenvironment differentiate into tumor-associated macrophages (TAMs), which adopt two distinct polarization states. Monocytes recruited into the tumor microenvironment can differentiate into tumor-associated macrophages (TAMs), which frequently exhibit immunosuppressive and tumor-promoting functions (13, 25). Elevated monocyte counts are generally regarded as a reflection of increased tumor burden and activation of a pro-tumorigenic microenvironment (13).

Dysregulated lipid metabolism is a prominent biological feature of ccRCC. The accumulation of intracellular lipid droplets represents a characteristic pathological feature of ccRCC (26). Dazhi Wang et al. proposed that ccRCC may exploit adipokine signaling pathways to sustain aggressive tumor growth (27). HDL-C plays important anti-inflammatory, antioxidative, and immunomodulatory roles beyond its classical function in cholesterol transport (28). Elevated HDL-C is generally associated with better overall health and confers multiple protective effects in cardiovascular disease, including anti-inflammatory, antithrombotic, and antioxidative properties (29). Accumulating evidence has demonstrated a close association between low HDL-C levels and increased risk of malignancy (30–32). HDL-C may represent a protective factor in ccRCC. Bo Hao et al. demonstrated that higher HDL-C levels were associated with improved overall survival and cancer-specific survival in patients with clear cell renal cell carcinoma (33).

Composite indices derived from peripheral blood cell counts and lipid metabolism parameters have gained increasing attention owing to their accessibility, low cost, and reproducibility. As composite biomarkers reflecting both systemic inflammation and lipid metabolic status, the neutrophil-to-HDL-C ratio (NHR) and monocyte-to-HDL-C ratio (MHR) have been reported to correlate closely with prognosis across multiple malignancies (34–37). Our research group has previously reported that apolipoprotein B (ApoB) (38)、low-density-lipoprotein-cholesterol (LDL-C) (39)、high-density lipoprotein cholesterol (HDL-C) (33)、C-reactive protein/albumin (CRP/Alb) ratio (40) serve as prognostic biomarkers for ccRCC. The C-reactive protein-albumin-lymphocyte (CALLY) index has also been validated as a biomarker for early-stage ccRCC (41). Combining inflammatory cells with HDL-C to construct ratio-based indices may more comprehensively capture the interplay between tumor-associated inflammation and metabolic dysregulation than either inflammatory cell counts or lipid parameters alone. This may partly explain why NHR and MHR showed more consistent prognostic discrimination than PHR and LHR in our cohort. Elevated NHR and MHR likely reflect enhanced pro-tumorigenic inflammation alongside attenuated protective lipid metabolism, collectively indicating a more unfavorable tumor-host state. Notably, not all ratios derived from circulating immune cells and HDL-C carry equivalent prognostic value. This discrepancy suggests that circulating hematological components may reflect distinct biological processes in tumor progression. Among these, neutrophils and monocytes appear to more accurately mirror the inflammatory and immune imbalance characteristic of ccRCC, suggesting that NHR and MHR may better capture the inflammatory-metabolic imbalance associated with ccRCC progression.

This study has several limitations. First, its retrospective single-center design may have introduced selection and information biases, limiting the generalizability of the findings. Second, the limited numbers of OS and CSS events may have reduced the precision and stability of the multivariable estimates. Third, only baseline preoperative hematological indices were assessed, and longitudinal changes in NHR and MHR during follow-up could not be evaluated. Residual confounding may also remain despite adjustment for multiple clinicopathological variables. In addition, the cutoff values for NHR and MHR were derived and evaluated in the same cohort, which may have introduced optimism bias despite bootstrap internal validation. CSS was analyzed using conventional Cox regression without accounting for non-cancer death as a competing event; therefore, the CSS results require cautious interpretation. Finally, although NHR and MHR were independently associated with survival, their addition to an already well-performing clinicopathological model produced only modest gains in discrimination and net benefit. External validation using independent cohorts and competing-risk methods is therefore needed before these biomarkers can be considered for routine clinical use.

## Conclusion

In conclusion, among the preoperative hematological indices evaluated in this study, NHR and MHR were more consistently associated with postoperative survival than PHR and LHR and remained independent prognostic factors after adjustment for established clinicopathological variables. However, the added prognostic value of NHR and MHR beyond conventional clinicopathological factors was limited. Further validation in independent multicenter cohorts, together with mechanistic studies, is needed before their clinical utility can be established.

## Data Availability

The raw data supporting the conclusions of this article will be made available by the authors, without undue reservation.

## Supplementary material

Supplementary Figure 1 Kaplan–Meier survival curves according to combined NHR–MHR stratification. Patients were categorized into four groups according to the predefined cutoff values of neutrophil-to-high-density lipoprotein cholesterol ratio (NHR) and monocyte-to-high-density lipoprotein cholesterol ratio (MHR) (NHR cutoff = 4.52; MHR cutoff = 0.49): low NHR + low MHR, high NHR + low MHR, low NHR + high MHR, and high NHR + high MHR. (A) Overall survival (OS) according to combined NHR–MHR groups. (B) Cancer-specific survival (CSS) according to combined NHR–MHR groups. Survival differences among groups were evaluated using the log-rank test.
