# Supplementary material for: Prognostic value of preoperative inflammatory cell-to-HDL cholesterol ratios in patients with non-metastatic clear cell renal cell carcinoma
Source: Front Oncol. 2026 Aug 12;16:1876997. doi: 10.3389/fonc.2026.1876997 (PMC13506302; doi:10.3389/fonc.2026.1876997)
Supplement: Supplementary file 1 [file Table1.docx]

**Supplementary Table S1. Bootstrap validation of cutoff stability and prognostic estimates for NHR and MHR**

| **Markers** | **Original cutoff** | **Bootstrap cutoff median (95% CI)** | **Original HR** | **Bootstrap HR median (95% CI)** |
| --- | --- | --- | --- | --- |
| NHR | 4.52 | 4.52 (4.00–5.13) | 4.57 | 4.83 (2.25–13.61) |
| MHR | 0.49 | 0.49 (0.34–0.69) | 4.33 | 4.88 (2.02–20.09) |

**Supplementary Table S2. Univariable Cox regression analysis according to combined NHR–MHR groups**

| **Groups** | **OS** | | **CSS** | |
| --- | --- | --- | --- | --- |
|  | **HR (95% CI)** | **P value** | **HR (95% CI)** | **P value** |
| Low NHR + Low MHR | Reference |  | Reference |  |
| High NHR + Low MHR | 8.47 (2.97–24.21) | <0.001 | 9.50 (3.24–27.89) | <0.001 |
| Low NHR + High MHR | 2.61 (0.92–7.42) | 0.072 | 2.95 (1.01–8.62) | 0.048 |
| High NHR + High MHR | 8.88 (4.26–18.50) | <0.001 | 9.83 (4.46–21.69) | <0.001 |

**Supplementary Table S3. Multivariable Cox regression analysis incorporating combined NHR–MHR groups**

| **Variables** | **OS** | | **CSS** | |
| --- | --- | --- | --- | --- |
|  | **HR (95% CI)** | **P value** | **HR (95% CI)** | **P value** |
| Age | 1.01 (0.98–1.04) | 0.452 | 1.01 (0.98–1.04) | 0.570 |
| Fuhrman grade |  | 0.289 |  | 0.331 |
| 1-2 | Reference |  | Reference |  |
| 3–4 | 1.51 (0.71-3.21) | 0.289 | 1.49 (0.67-3.32) | 0.331 |
| T stage |  | <0.001 |  | <0.001 |
| T1a | Reference |  | Reference |  |
| T1b-T2 | 4.63 (1.62–13.21) | 0.004 | 5.72 (1.65-19.79) | 0.006 |
| T3-4 | 17.65 (5.08–61.33) | <0.001 | 23.21(5.61-96.09) | <0.001 |
| Combined NHR–MHR group |  | <0.001 |  | <0.001 |
| Low NHR + Low MHR | Reference |  |  |  |
| High NHR + Low MHR | 5.51 (1.78–17.05) | 0.003 | 5.58 (1.74–17.86) | 0.004 |
| Low NHR + High MHR | 3.22 (1.09–9.48) | 0.034 | 3.60 (1.18–10.94) | 0.024 |
| High NHR + High MHR | 7.20 (3.25–15.96) | <0.001 | 6.86 (2.88–16.31 | <0.001 |
